# Assignment of Absolute Configuration of a New Hepatoprotective Schiartane-Type Nortriterpenoid Using X-ray Diffraction

**DOI:** 10.3390/molecules22010065

**Published:** 2017-01-02

**Authors:** Xiaojuan Wang, Frank R. Fronczek, Jiabao Chen, Jiabao Liu, Daneel Ferreira, Shuai Li, Mark T. Hamann

**Affiliations:** 1 State Key Laboratory of Bioactive Substances and Functions of Natural Medicines, Institute of Materia Medica, Chinese Academy of Medical Sciences and Peking Union Medical College, Beijing 100050, China; xiaojuan2013@outlook.com (X.W.); cjb427@hotmail.com (J.C.); ljb_5021@hotmail.com (J.L.); 2 Department of Drug Discovery and Biomedical Sciences, College of Pharmacy, Medical University of South Carolina, Charleston, SC 29425, USA; 3 Department of Chemistry, Louisiana State University, Baton Rouge, LA 70803, USA; ffroncz@lsu.edu; 4 Department of BioMolecular Sciences, Division of Pharmacognosy, and Research Institute of Pharmaceutical Sciences, School of Pharmacy, University of Mississippi, University, MS 38677-1848, USA; dferreir@olemiss.edu

**Keywords:** schiartane-type nortriterpenoid, X-ray, absolute configuration, hepatoprotective

## Abstract

A new schiartane-type nortriterpenoid, micrandilactone H was isolated from *Kadsura longipedunculata* Finet et Gagnep. Its 2D (two dimension) structure was elucidated by NMR spectroscopic analysis, and it is similar to that of Kadnanolactones H and the absolute configuration was established through X-ray diffraction and ECD data analysis. This represents the first complete assignment of the absolute configuration of a schiartane-type nortriterpenoid by X-ray diffraction and the ECD method. Micrandilactone H showed moderate hepatoprotective activity against *N*-acetyl-*p*-aminophenol (APAP)-induced toxicity in HepG2 cells with cell survival rates of 56.84% at 10 μM.

## 1. Introduction

The roots and stems of plants of the genus *Kadsura*, have been used in traditional Chinese medicines (TCM) to treat blood disease, remove stasis and relieve pain. These plants are widely distributed in the southwest of China. Phytochemical studies showed that the principal bioactive constituents of the genus *Kadsura* are lignans and triterpenoids. The lignans include dibenzocyclooctadiene lignans, *spiro*-benzofuran-type dibenzocyclooctadiene lignans, cyclolignans, neolignans and simple lignans. The triterpenoids include lanostane-type, cycloartane-type and nortriterpenoids. Nortriterpenoids are a structurally intriguing group of highly oxygenated, polycyclic, fused heterocyclic natural products produced by the plants of the genera *Schisandra* and *Kadsura*. Some of these compounds showed promising anti-HIV (human immunodeficiency virus) bioactivities. The C29 type is the most common and can be divided into five classes including schiartane, 18(13→14)-abeo-schiartane, schisanartane, preschisanartane and wuweiziartane [1]. Their planar structures and relative configurations have been successfully elucidated by modern NMR techniques. Thus far, the side chain of only one member of this class has been successfully assigned unambiguously using X-ray diffraction combined with the modified Mosher method. [2] Assignment of the absolute configuration is critical since it influences the bioactivity and is essential for future efforts focused on synthesis and medicinal chemistry. Owing to the unique structures and activities there has been an escalating interest in their discovery, total synthesis, biological activities and biosynthesis studies [1].

*Kadsura longipedunculata* belongs to the family Schisandraceae [2]. The roots have been used in Chinese folk medicine to treat irregular menstruation, gastroenteritis, fractures and rheumatoid arthritis [2]. In order to identify new natural compounds with interesting bioactivities, we performed a phytochemical study on the roots of *K. longipedunculata* that lead to the identification of a new C29 triterpenoid dilactone, micrandilactone H. Micrandilactone H showed moderate hepatoprotective activity against *N*-acetyl-*p*-aminophenol (APAP)-induced toxicity in HepG2 (human hepatocellular liver carcinoma cell line) cells with cell survival rates of 56.84% at 10 μM. This is the first report of the full stereochemistry assignment of a schiartane-type nortriterpenoid via X-ray diffraction and it is supported by ECD and NMR spectroscopic data.

## 2. Results

**Figure 1:**
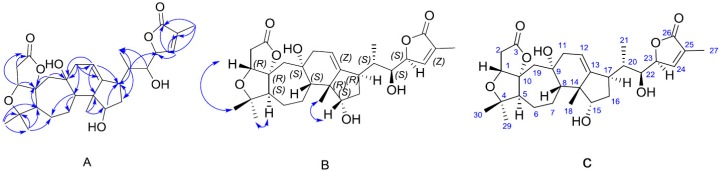
Key HMBC (**A**), NOESY (**B**) correlations of micrandilactone H (**C**).

Micrandilactone H was isolated as colorless crystals, and gave an [M + Na]^+^ ion at *m*/*z* 539.2637 in the HRESIMS, consistent with a molecular formula of C_29_H_40_NaO_8_ (calcd 539.2615), indicating nine indices of hydrogen deficiency. The ^13^C and HSQC NMR spectra (Table 1 and Table S7) revealed two lactone carbonyls resonating at δ_C_ 175.9 and 175.1; two olefinic functionalities with four carbon resonances at δ_C_ 147.7, 120.9, 148.9 and 130.5; four sp^3^ non-protonated carbons (three oxygenated) resonances at δ_C_ 99.7, 85.2, 70.8 and 52.8; eight sp^3^ methine (three oxygenated) resonances at δ_C_ 82.5, 59.6, 58.8, 77.1, 43.3, 44.3, 74.9 and 83.0 ppm; six sp^3^ methylene resonances at δ_C_ 46.0, 42.7, 40.4, 36.9, 28.7, and 25.3; and five methyl resonances at δ_C_ 29.9, 26.6, 23.5, 16.1, and 10.9. The ^1^H-NMR spectra revealed an ABX spin system, with δ_H_ 4.27 (d, *J* = 4.5 Hz), 2.94 (dd, *J* = 18.0, 4.5 Hz) and 2.72 (d, *J* = 18.0 Hz), which were assigned to H-1, H-2α and H-2β, respectively, together with four methyl singlets at δ_H_ 1.83 (3H, s), 1.27 (3H, s), 1.14 (3H, s), 1.12 (3H, s); one methyl doublet at δ_H_ 1.34 (3H, d, *J* = 7.0 Hz); two olefinic protons (δ_H_ 7.23, 5.76); four methine protons (δ_H_ 5.27, 4.00 and 3.31); and several methylene protons between δ_H_ 1.22 and 2.80. According to these data, micrandilactone H is a schiartane-type nortriterpenoid whose structure is similar to micrandilactone C, except for an extra double bond, one less hydroxy group and the different location of the C-18 methyl group [3]. The HMBC cross-peaks of H-18 with C-8, C-13, C-14 and C-15, H-11 with the C-8, C-9, C-12, C-13 and C-19, and H-12 with C-9, C-14 and C-17 (Figure S8) indicated that Me-18 was connected at C-14 and the double bond was located at C-12 and C-13. Thus, the 2D (two dimension) structure of micrandilactone H was established as shown in Figure 1.

**Figure 2:**
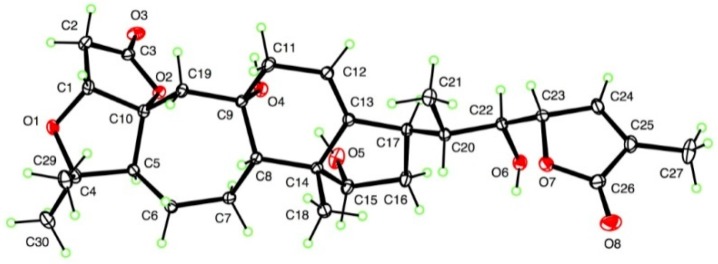
ORTEP drawing of micrandilactone H.

The absolute configuration was unambiguously determined as (1*R*, 5*S*, 8*S*, 9*S*, 10*R*, 14*R*, 15*S*, 17*R*, 20*S*, 22*S*, 23*S*) by X-ray crystallographic analysis (Figure 2) based on resonant scattering of only light atoms in both Mo and Cu radiations. These results matched the assignment of the (23*S*) absolute configuration based on the negative Cotton effect at 214 nm in the ECD spectrum [4]. The molecular structure features an intramolecular hydrogen bond between the 15- and 9-OH groups. However, the 22-OH group does not form an intramolecular hydrogen bond with the adjacent lactone carbonyl, instead forming an intermolecular hydrogen bond with the C-3 lactone carbonyl. 

Micrandilactone H was evaluated in vitro for its hepatoprotective activity against APAP-induced toxicity in HepG2 cells, using the hepatoprotective activity drug bicyclol as the positive control. Micrandilactone H affected a cell survival rate of 56.84% (bicyclol, 49.00%) at 10 μM when added into the resuscitated HepG2 cells incubated with APAP for 48 h.

## 3. Materials and Methods

### 3.1. General Experimental Procedures

Optical rotations were measured on a JASCO P-2000 polarimeter (Jasco Inc., Easton, MD, USA) and UV spectra with a JASCO V-650 spectrophotometer (Jasco Inc., Easton, MD, USA). ECD spectra were recorded on a JASCO J-815 spectrometer (Jasco Inc., Easton, MD, USA). IR spectra were recorded on a Nicolet 5700 spectrometer (Thermo Electron Corporation, Madison, WI, USA) using an FT-IR microscope transmission method. The 1H- and ^13^C-NMR spectra were recorded on INOVA-500 (Varian, Inc., Palo Alto, CA, USA) and BRUKER AV500-III spectrometers (Bruker, Billerica, MA, USA). The standard pulse sequences were used for each 2D measurement. HRESIMS data were acquired an Agilent 6520 Accurate-Mass Q-Tof LC/MS spectrometer (Agilent Technologies, Waldbronn, Germany). Analytical reversed-phase HPLC was performed on a COSMOSIL 5C18-PAQ Waters column (4.6 i.d. × 250 mm, Waters, Nacalai, San Diego, CA, USA) eluted with water/methanol (flow rate, 1 mL/min; 220 nm UV detection) at room temperature. Preparative reversed-phase HPLC was performed on a COSMOSIL 5C18-PAQ Waters column (250 × 10 mm, 5 μm) at room temperature. Column chromatography was performed on silica gel (40–63 μm; Silicyle, Quebec City, QC, Canada), C18 120 Å reversed-phase silica gel (RP-18; 50μm; Silicycle) and Sephadex LH-20 (GE Healthcare Bio-Science AB, Uppsala, Sweden). Fractions were monitored by TLC and spots were visualized by heating silica gel plates sprayed with 10% H_2_SO_4_ in EtOH.

### 3.2. Plant Material

The roots of *K. longipedunculata* were collected in the Jiujiang County of Jiangxi Province, China, in March 2010, and identified by Ce-Ming Lin, Institute of Biology Resources, Jiangxi Academy of Science. A voucher specimen (ID-S-2428) is deposited in the herbarium of the Institute of Materia Medica (IMM), Chinese Academy of Medical Science (CAMS) and Peking Union Medical College (PUMC).

### 3.3. Extraction and Isolation 

The air-dried and powered roots (34.0 kg) were percolated with 95% aqueous ethanol (4 × 12 L) at room temperature and the solvent removed in vacuo to yield a residue (2400 g). The residue was subjected to silica gel (200–300 mesh, 2400 g) column chromatography, eluting with a petroleum ether/acetone gradient system (100:1, 50:1, 10:1, 5:1, 3:1, 1:1), acetone and 80% aqueous ethanol, to give fractions 1–15. Fraction 14 was partitioned between water and EtOAc. The EtOAc extract (60 g) was chromatographed on an ODS column eluted with gradient water/methanol (4:1, 3:2, 2:3, 1:4) to yield five fractions A–E. Fraction D was chromatographed on Sephadex LH-20 (eluted with chloroform/methanol) and gave five subfractions 1–5. Fraction D1 (1.7 g) was subjected to silica gel (200–300 mesh, 3 g) column chromatography, eluting with chloroform/methanol gradient system (4:1 to 1:4) and give nine subfractions, D1.1–D1.9. Fraction D1.3 (365 mg) purified by semi-preparative HPLC (46% methanol/water), to give micrandilactone H (3 mg). Micrandilactone H (3 mg) was dissolved in 1mL of methanol, 0.5 mL of water was added, the sample was kept at room temperature, and the single crystal was generated. 

*Micrandilactone H*: Colorless crystals; [*α*]${}_{D}^{20}$ 3 (*c* 0.4, methanol); UV (methanol) λ_max_ (log ε) 211 (2.29) nm; ECD (methanol) [*θ*] (nm) −12.0 × 10^3^ (214). IR (KBr) *ν*_max_ 3525, 3468, 3386, 2973, 2935, 1747, 1066, 923, 867 cm^−1^. ESIMS (*m*/*z*): 539 [M + Na]^+^;HRESIMS (*m*/*z*): 539.2637 [M + Na]^+^ (calcd 539.2615 for C_29_H_40_NaO_8_).

### 3.4. X-Ray Crystallographic Analysis of Micrandilactone H

C_29_H_40_O_8_, M = 516.61, monoclinic, space group P21, a = 10.8666 (16) Å, b = 10.3174 (16) Å, c = 12.6133 (19) Å, β = 113.828 (7)^o^, V = 1293.6 (3) Å^3^, Z = 2, d = 1.326 g cm^−3^. Intensity data were measured at T = 90 K on a Bruker Kappa APEX-II DUO diffractometer, including both Mo Kα and Cu Kα datasets. The total number of reflections measured with Mo radiation to θ_max_ = 36.4^o^ was 21,401, yielding 10,250 independent data, of which 9425 were observed with F2 > 2σ(F2), Rint = 0.027. The structure was solved using SHELXS-97 and refined [5] by full-matrix least-squares using SHELXL-2014/7. R = 0.036, wR(F2) = 0.095 for all data and 348 refined parameters. The Flack parameter based on 3290 quotients [6] was 0.0(2), indicating the absolute configuration shown in Figure 2, CCDC 1520861. To confirm the absolute configuration, Cu data from a microfocus source was measured using the same crystal to a maximum θ value of 69.1°, yielding 20,583 total data and 4561 independent data, Rint = 0.041. Refinement yielded R = 0.026, wR(F2) = 0.066, and Flack parameter 0.01(4) for 2044 quotients, giving a much stronger indication of an absolute configuration of (1*R*, 5*S*, 8*S*, 9*S*, 10*R*, 14*R*, 15*S*, 17*R*, 20*S*, 22*S*, 23*S*), CCDC 1520862.

### 3.5. Hepatoprotective Activity Assay

Micrandilactone H was tested for hepatoprotective activity against APAP-induced toxicity in HepG2 cells by means of the published MTT method [7].

## Figures and Tables

**Table 1:** ^1^H- and ^13^C-NMR data for micrandilactone H in pyridine-*d*_5_ using an INOVA-500 MHz spectrometer.

Position	δ_H_	δ_C_
1	4.27 (d, 4.5)	82.5
2α	2.72 (d, 18.0)	36.9
2β	2.94 (dd, 18.0, 4.5)	
3		175.9
4		85.2
5	2.57 (dd, 13.0, 4.0)	59.6
6α	1.69 (overlapped)	28.7
6β	1.31 (overlapped)	
7α	2.37 (m)	25.3
7β	2.07 (overlapped)	
8	1.72 (overlapped)	58.8
9		70.8
10		99.7
11α	2.35 (m)	42.7
11β	2.26 (m)	
12α	5.76 (d, 7.0)	120.9
12β		
13		147.7
14		52.8
15	4.00 (s)	77.1
16α	2.30 (m)	40.4
16β	2.11 (dd, 8.5, 3.0)	
17	3.31 (m)	43.3
18	1.12 (s)	26.6
19α	2.15 (d, 15.0)	46.0
19β	2.02 (d, 15.0)	
20	2.21 (m)	44.3
21	1.34 (d, 7.0)	16.1
22	4.00 (s)	74.9
23	5.27 (br, s)	83.0
24	7.23 (s)	148.9
25		130.5
26		175.1
27	1.83(s)	10.9
29	1.27(s)	29.9
30		23.5

## Supplementary Materials

^1^H-NMR, ^13^C-NMR, HMBC, HSQC, ROESY, ECD and HRESIMS data for micrandilactone H and the detailed experimental conditions for hepatoprotective assay are available free of charge via the Internet at http://www.mdpi.com/1420-3049/22/1/65/s1. CCDC 1520861-1520862 contains the supplementary crystallographic data for this paper. These data can be obtained free of charge via http://www.ccdc.cam.ac.uk/conts/retrieving.html (or from the CCDC, 12 Union Road, Cambridge CB2 1EZ, UK; Fax: +44 1223 336033; E-mail: deposit@ccdc.cam.ac.uk.
