# Supplementary material for: Assignment of Absolute Configuration of a New Hepatoprotective Schiartane-Type Nortriterpenoid Using X-ray Diffraction
Source: Molecules. 2017 Jan 2;22(1):65. doi: 10.3390/molecules22010065 (PMC6155901; doi:10.3390/molecules22010065)
Supplement: Supplementary file 1 [file molecules-22-00065-s001.pdf]

Supplementary Materials: Assignment of Absolute Configuration of a New Hepatoprotective Schiartane-Type Nortriterpenoid Using X-Ray Diffraction

Xiaojuan Wang, Frank R. Fronczek, Jiabao Chen, Jiabao Liu, Daneel Ferreira, Shuai Li and Mark T. Hamann

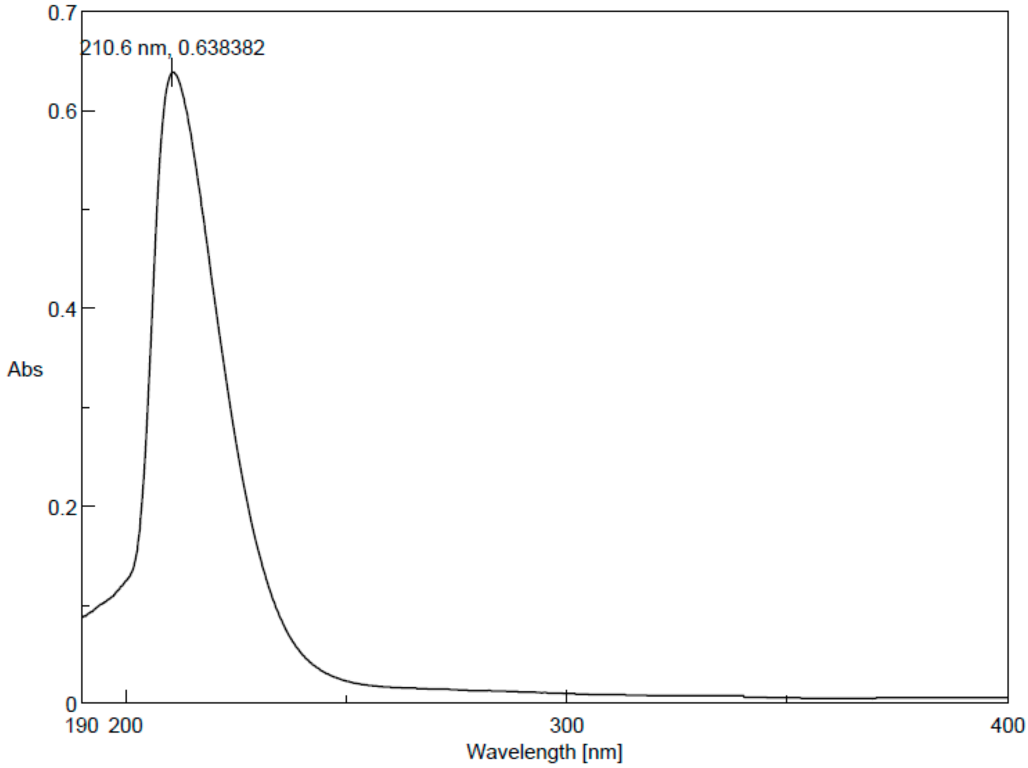

Figure S1. The UV spectrum of micrandilactone H.

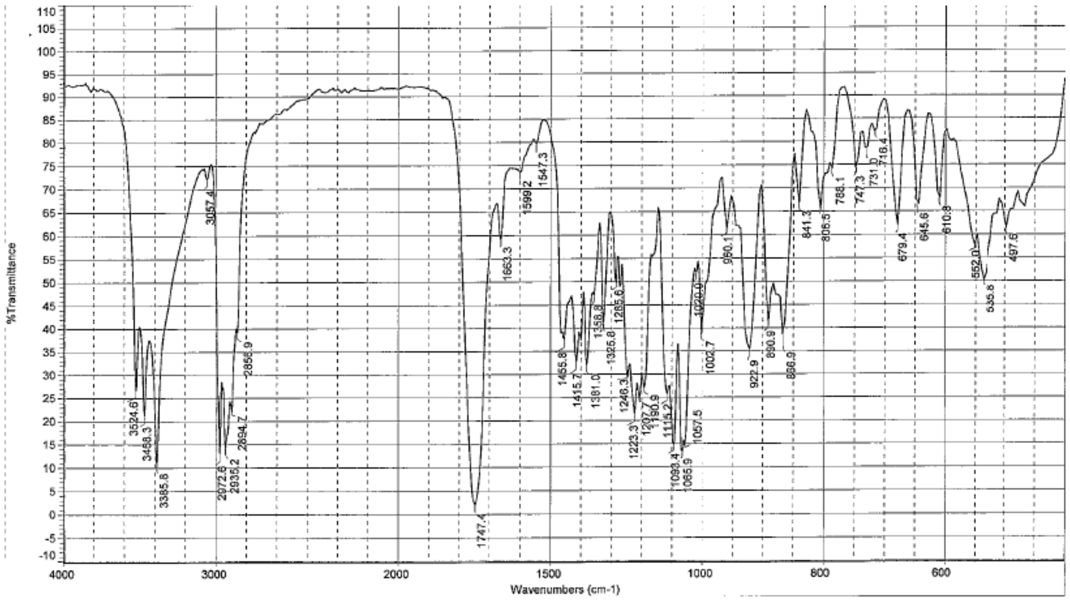

Figure S2. The IR spectrum of micrandilactone H.

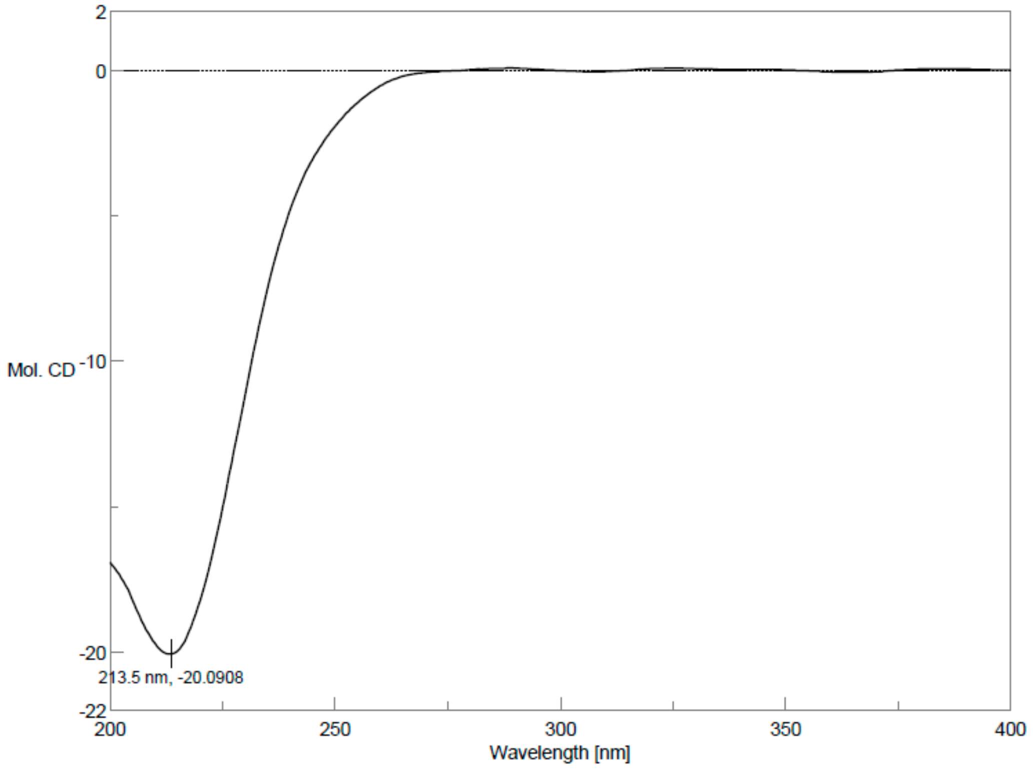

Figure S3. The ECD spectrum of micrandilactone H.

m/z	Ion	Formula	Abundance
517.2806	(M+H)+	C29 H41 O8	1969917.5

Best	Formula (M)	Ion Formula	Score	Cross Score	Mass	Calc Mass	Calc m/z	Diff (ppm)	Abs Diff (ppm)	Mass Match	Abund Match	Spacing Match	DBE
✓	C29 H40 O8	C29 H41 O8	99.79		516.2734	516.2723	517.2796	-2	2	99.86	99.55	99.92	10

m/z	Ion	Formula	Abundance
539.2637	(M+Na)+	C29 H40 Na O8	876727.8

Best	Formula (M)	Ion Formula	Score	Cross Score	Mass	Calc Mass	Calc m/z	Diff (ppm)	Abs Diff (ppm)	Mass Match	Abund Match	Spacing Match	DBE
✓	C29 H40 O8	C29 H40 Na O8	99.68		516.2744	516.2723	539.2615	-4.1	4.1	99.47	99.85	99.91	10

Figure S4. The HR-ESI-MS spectrum of micrandilactone H.

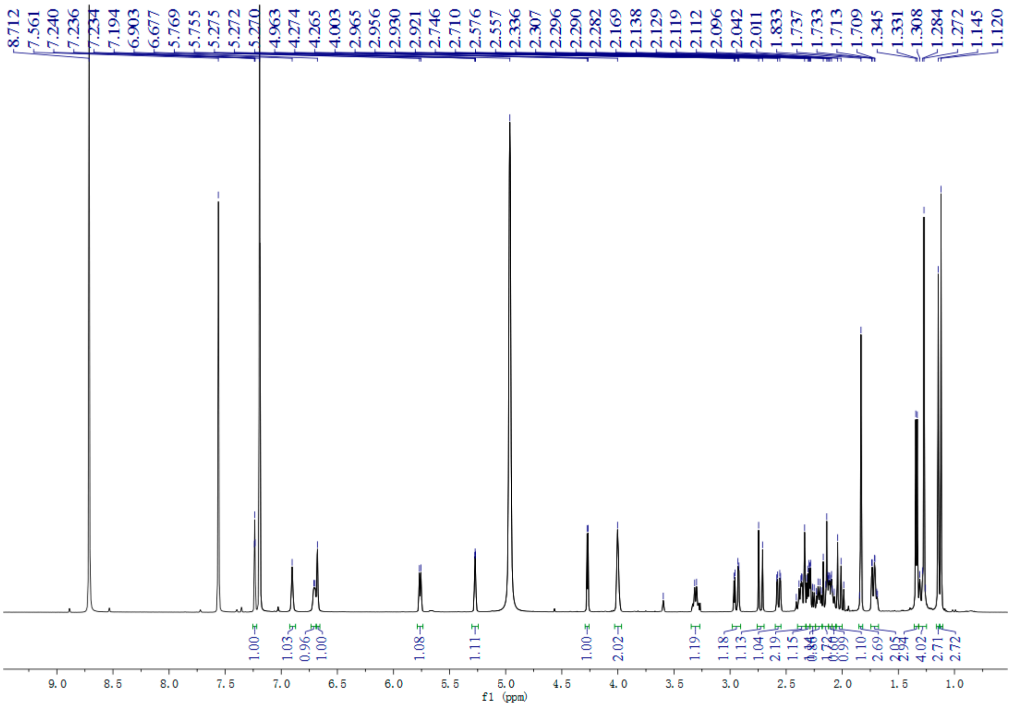

Figure S5. The ^{1}H-NMR spectrum of micrandilactone H.

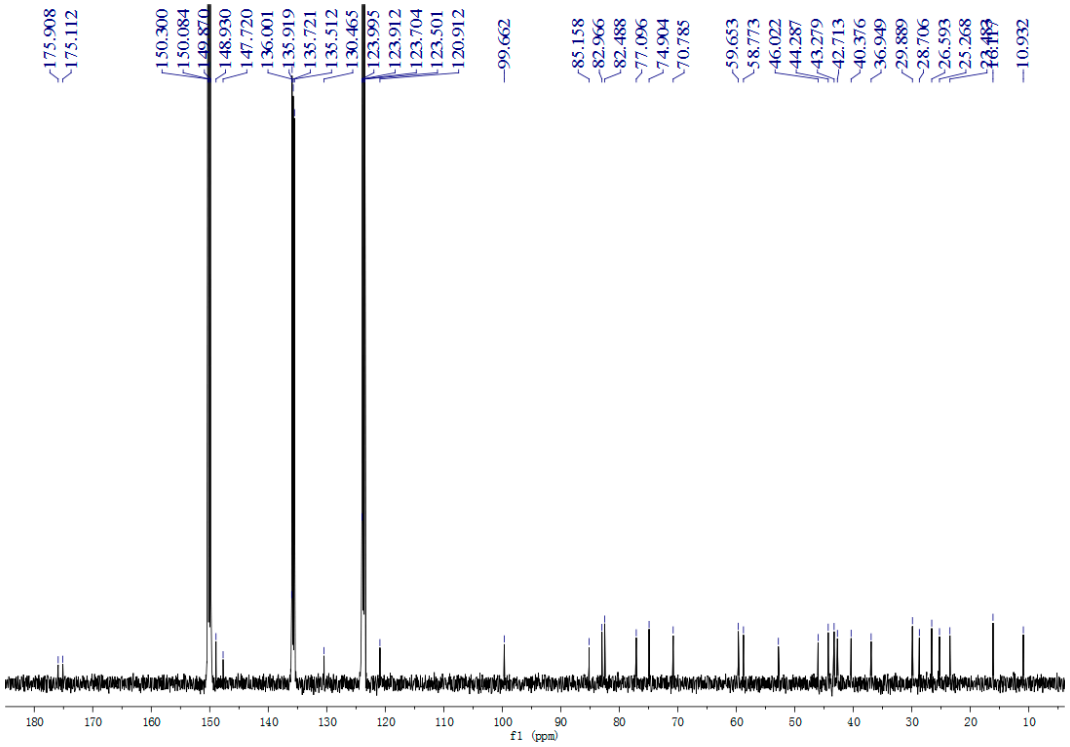

Figure S6. The ^{13}C-NMR spectrum of micrandilactone H.

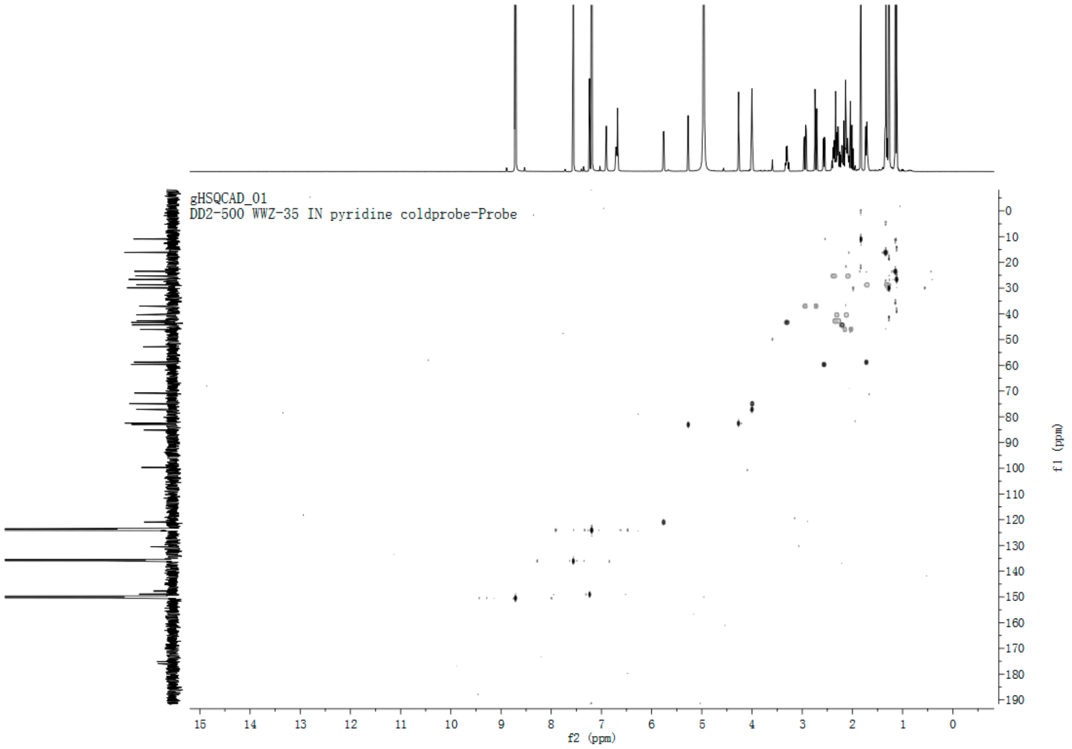

Figure S7. The HSQC spectrum of micrandilactone H.

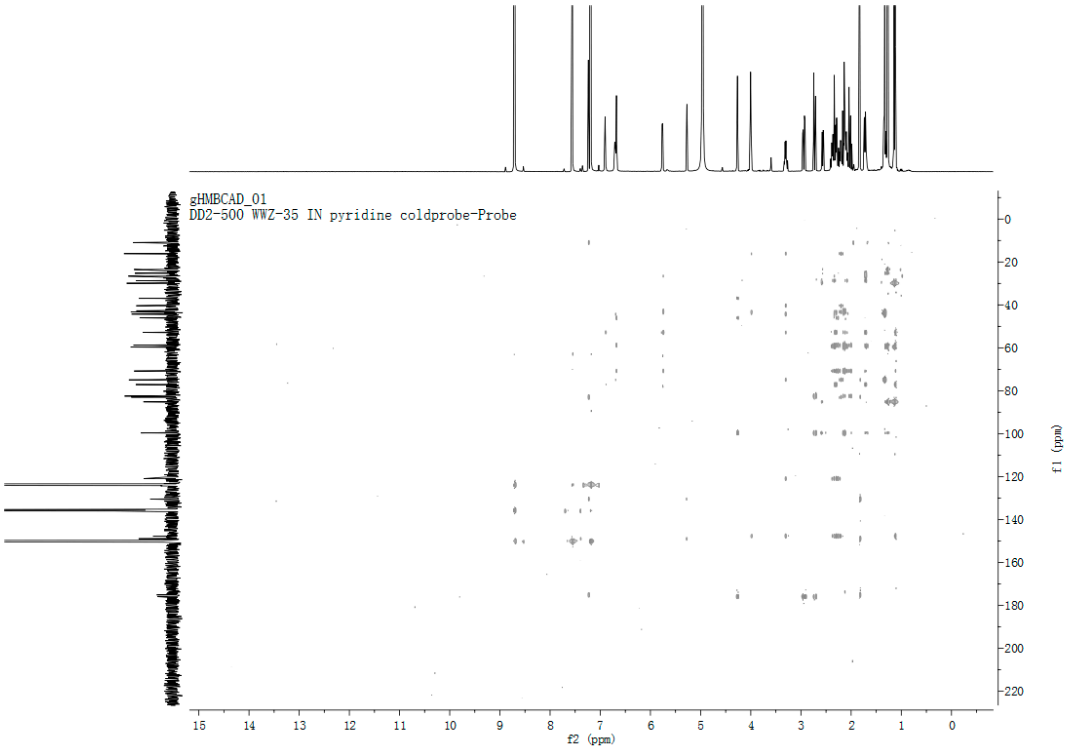

Figure S8. The HMBC spectrum of micrandilactone H.

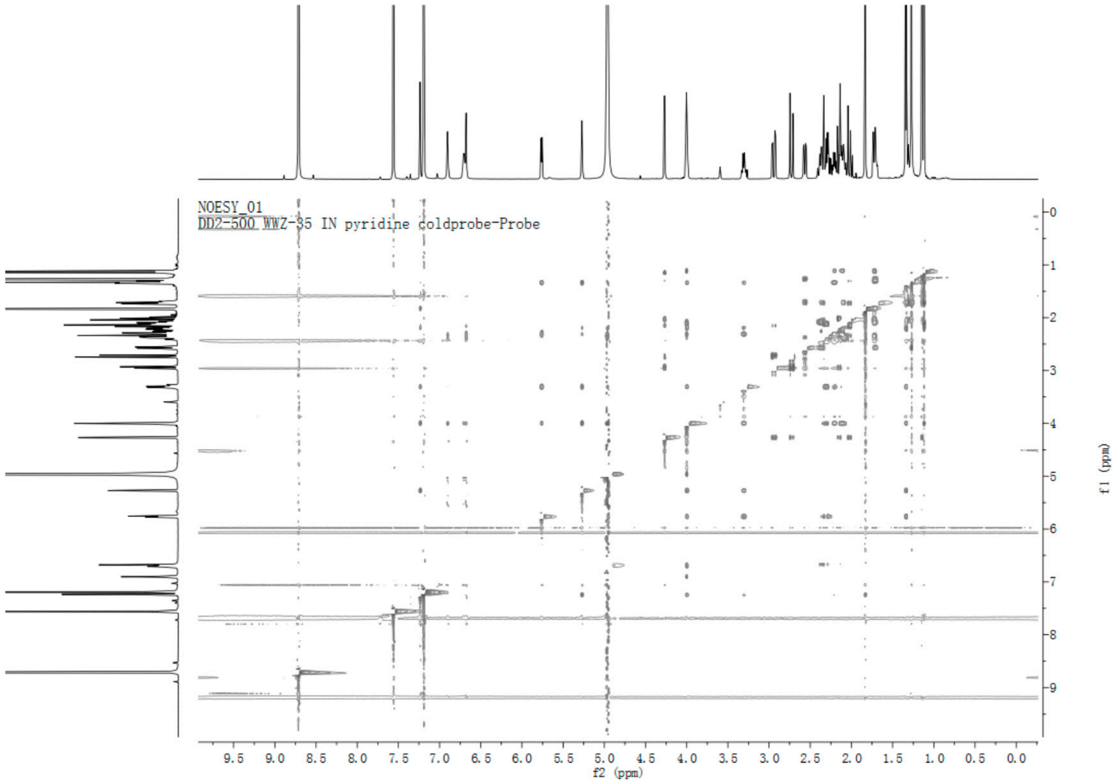

Figure S9. The NOESY spectrum of micrandilactone H.

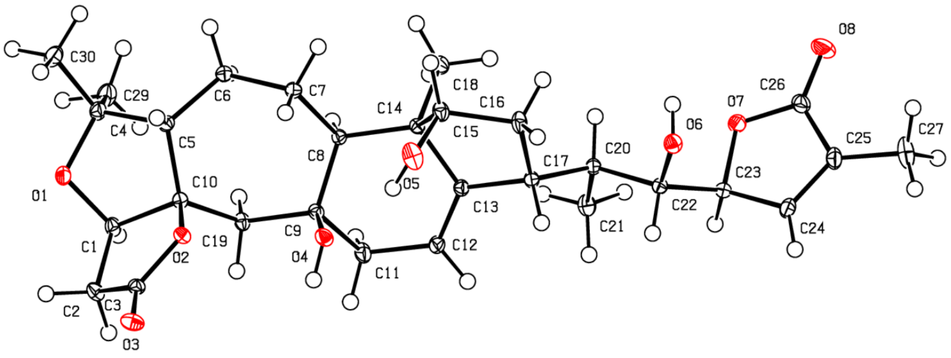

Figure S10. ORTEP plot of the molecular structure of micrandilactone H. Red = oxygen; grey = carbon; white = hydrogen.

Table S1. Crystal data and structure refinement for micrandilactone H.

Identification Code	Micrandilactone H.
Empirical formula	$C_{29}H_{40}O_8$
Formula weight	516.61
Temperature	90 K
Wavelength	0.71073 Å
Crystal system	Monoclinic
Space group	$P2_1$
	a = 10.8666 (16) Å, α = 90°
Unit cell dimensions	b = 10.3174 (16) Å, β = 113.828(7)°
	c = 12.6133 (19) Å, γ = 90°.
Volume	1293.6 (3) $Å^3$
Z	2
Density (calculated)	1.326 mg/m^3
Absorption coefficient, μ	0.10 mm^{-1}
F(000)	556.0
Crystal size	0.33 × 0.29 × 0.15 mm
Theta range for data collection	1.8° to 36.4°
Index ranges	−18 ≤ h ≤ 16, −17 ≤ k ≤ 13, −20 ≤ l ≤ 20
Reflections collected	21,401
Independent reflections	10,252
Completeness to theta = 25.2°	0.999
Absorption correction	Multi-scan
Max. and min. transmission	0.986 and 0.934
Refinement method	Full-matrix least-squares on F^2
Data/restraints/parameters	10252/1/348
Goodness-of-fit on F^2	1.04
Final R [I>2sigma(I)]	0.036
R (all data)	0.041
Absolute structure parameter	0.0(2)
Extinction coefficient	none
Largest diff. peak and hole	0.40, −0.23 $eÅ^{-3}$

Table S2. Fractional atomic coordinates and isotropic or equivalent isotropic displacement parameters (Å²) for micrandilactone H.

	x	*y*	*z*	U_{iso}*/U_{eq}
O1	0.72536 (8)	0.52140 (10)	0.44744 (7)	0.01375 (17)
O2	0.46412 (8)	0.63965 (8)	0.44877 (7)	0.00996 (15)
O3	0.34881 (9)	0.69061 (9)	0.26348 (8)	0.01523 (17)
O4	0.32344 (9)	0.60775 (10)	0.58935 (8)	0.01314 (16)
H4O	0.2667 (19)	0.583 (2)	0.5228 (17)	0.020 *
O5	0.32670 (11)	0.77748 (10)	0.75508 (9)	0.01838 (19)
H5O	0.307 (2)	0.721 (2)	0.7022 (18)	0.028*
O6	0.11200 (9)	0.66578 (9)	1.04811 (8)	0.01346 (16)
H6O	0.1822 (19)	0.664 (2)	1.1052 (17)	0.020*
O7	0.14669 (8)	0.43545 (9)	1.18981 (7)	0.01370 (17)
O8	0.17721 (11)	0.47960 (13)	1.37233 (9)	0.0267 (2)
C1	0.59976 (11)	0.46515 (12)	0.43314 (10)	0.0118 (2)
H1	0.6069	0.3695	0.4472	0.014*
C2	0.49446 (12)	0.49921 (13)	0.31467 (10)	0.0130 (2)
H2A	0.5366	0.5176	0.2598	0.016*
H2B	0.4290	0.4277	0.2833	0.016*
C3	0.42705 (11)	0.61851 (12)	0.33498 (10)	0.01077 (19)
C4	0.79711 (11)	0.54304 (13)	0.57031 (10)	0.0128 (2)
C5	0.68697 (11)	0.60346 (12)	0.60447 (10)	0.01039 (18)
H5A	0.6805	0.6973	0.5829	0.012*
C6	0.71704 (11)	0.59738 (13)	0.73337 (10)	0.0129 (2)
H6A	0.7987	0.6494	0.7762	0.015*
H6B	0.7373	0.5064	0.7596	0.015 *
C7	0.60256 (11)	0.64662 (13)	0.76546 (10)	0.0123 (2)
H7A	0.6423	0.6862	0.8436	0.015 *
H7B	0.5523	0.7152	0.7099	0.015 *
C8	0.50358 (11)	0.53999 (11)	0.76490 (9)	0.00924 (18)
H8	0.5609	0.4621	0.7986	0.011 *
C9	0.40725 (11)	0.49866 (12)	0.64283 (9)	0.00970 (18)
C10	0.55634 (10)	0.53822 (11)	0.52012 (9)	0.00915 (18)
C11	0.31924 (13)	0.38596 (13)	0.65300 (11)	0.0154 (2)
H11A	0.2524	0.3625	0.5748	0.018 *
H11B	0.3767	0.3092	0.6860	0.018 *
C12	0.24725 (12)	0.42141 (13)	0.72826 (10)	0.0136 (2)
H12	0.1620	0.3834	0.7126	0.016 *
C13	0.29837 (11)	0.50405 (12)	0.81600 (9)	0.01010 (18)
C14	0.43376 (11)	0.56952 (12)	0.84893 (9)	0.00931 (18)
C15	0.39495 (12)	0.71139 (12)	0.86178 (10)	0.0126 (2)
H15	0.4768	0.7612	0.9117	0.015 *
C16	0.29873 (13)	0.69653 (13)	0.92437 (11)	0.0139 (2)
H16A	0.2343	0.7696	0.9045	0.017 *
H16B	0.3504	0.6950	1.0094	0.017 *
C17	0.22296 (11)	0.56646 (12)	0.88195 (9)	0.01036 (19)
H17	0.1307	0.5882	0.8237	0.012 *
C18	0.53056 (12)	0.52340 (14)	0.97031 (10)	0.0147 (2)
H18A	0.4878	0.5348	1.0248	0.022 *
H18B	0.6136	0.5745	0.9964	0.022 *
H18C	0.5519	0.4316	0.9669	0.022 *
C19	0.48074 (11)	0.44821 (11)	0.56867 (10)	0.01039 (18)

Table S2. *Cont.*

	x	*y*	*z*	U_{iso}*/U_{eq}
H19A	0.5461	0.3822	0.6158	0.012 *
H19B	0.4129	0.4019	0.5018	0.012 *
C20	0.20562 (11)	0.48329 (12)	0.97848 (9)	0.01012 (18)
H20	0.2892	0.4925	1.0510	0.012 *
C21	0.18522 (13)	0.33929 (13)	0.94771 (11)	0.0144 (2)
H21A	0.1836	0.2905	1.0138	0.022 *
H21B	0.2592	0.3079	0.9290	0.022 *
H21C	0.0997	0.3271	0.8806	0.022 *
C22	0.08720 (11)	0.53849 (12)	1.00190 (9)	0.01094 (19)
H22	0.0099	0.5456	0.9246	0.013 *
C23	0.03925 (11)	0.45074 (13)	1.07598 (10)	0.01171 (19)
H23	0.0130	0.3641	1.0380	0.014 *
C24	-0.07391 (12)	0.50547 (14)	1.10001 (11)	0.0148 (2)
H24	-0.1599	0.5266	1.0421	0.018 *
C25	-0.03865 (13)	0.52078 (14)	1.21338 (11)	0.0157 (2)
C26	0.10340 (13)	0.47907 (14)	1.27055 (10)	0.0158 (2)
C27	-0.11622 (17)	0.56715 (18)	1.28035 (14)	0.0271 (3)
H27A	-0.2000	0.6079	1.2274	0.041 *
H27B	-0.0623	0.6305	1.3384	0.041 *
H27C	-0.1372	0.4935	1.3192	0.041 *
C29	0.85235 (13)	0.41378 (14)	0.63016 (12)	0.0169 (2)
H29A	0.7777	0.3590	0.6279	0.025 *
H29B	0.9136	0.4297	0.7110	0.025 *
H29C	0.9012	0.3700	0.5900	0.025 *
C30	0.91182 (12)	0.63674 (14)	0.58623 (11)	0.0168 (2)
H30A	0.9803	0.5926	0.5674	0.025 *
H30B	0.9520	0.6665	0.6669	0.025 *
H30C	0.8769	0.7114	0.5347	0.025 *

Table S3. Bond lengths (Å) and angles (°) for micrandilactone H.

O1—C1	1.4259 (15)	C12—C13	1.3281 (17)
O1—C4	1.4433 (15)	C12—H12	0.9500
O2—C3	1.3426 (14)	C13—C14	1.5164 (16)
O2—C10	1.4767 (14)	C13—C17	1.5261 (16)
O3—C3	1.2125 (15)	C14—C18	1.5414 (16)
O4—C9	1.4334 (15)	C14—C15	1.5498 (18)
O4—H4O	0.85 (2)	C15—C16	1.5514 (17)
O5—C15	1.4212 (16)	C15—H15	1.0000
O5—H5O	0.85 (2)	C16—C17	1.5521 (18)
O6—C22	1.4178 (16)	C16—H16A	0.9900
O6—H6O	0.81 (2)	C16—H16B	0.9900
O7—C26	1.3595 (16)	C17—C20	1.5619 (16)
O7—C23	1.4474 (14)	C17—H17	1.0000
O8—C26	1.2081 (15)	C18—H18A	0.9800
C1—C2	1.5114 (17)	C18—H18B	0.9800
C1—C10	1.5533 (16)	C18—H18C	0.9800
C1—H1	1.0000	C19—H19A	0.9900
C2—C3	1.5062 (17)	C19—H19B	0.9900
C2—H2A	0.9900	C20—C21	1.5289 (19)
C2—H2B	0.9900	C20—C22	1.5410 (16)
C4—C30	1.5253 (19)	C20—H20	1.0000
C4—C29	1.5305 (19)	C21—H21A	0.9800

Table S3. *Cont.*

C4—C5	1.5567 (16)	C21—H21B	0.9800
C5—C6	1.5247 (16)	C21—H21C	0.9800
C5—C10	1.5427 (16)	C22—C23	1.5355 (16)
C5—H5A	1.0000	C22—H22	1.0000
C6—C7	1.5415 (17)	C23—C24	1.4912 (17)
C6—H6A	0.9900	C23—H23	1.0000
C6—H6B	0.9900	C24—C25	1.3320 (18)
C7—C8	1.5367 (17)	C24—H24	0.9500
C7—H7A	0.9900	C25—C26	1.4794 (19)
C7—H7B	0.9900	C25—C27	1.4927 (18)
C8—C9	1.5317 (15)	C27—H27A	0.9800
C8—C14	1.5625 (15)	C27—H27B	0.9800
C8—H8	1.0000	C27—H27C	0.9800
C9—C11	1.5435 (17)	C29—H29A	0.9800
C9—C19	1.5451 (15)	C29—H29B	0.9800
C10—C19	1.5228 (16)	C29—H29C	0.9800
C11—C12	1.4989 (17)	C30—H30A	0.9800
C11—H11A	0.9900	C30—H30B	0.9800
C11—H11B	0.9900	C30—H30C	0.9800
C1—O1—C4	105.84 (9)	O5—C15—C14	114.46 (10)
C3—O2—C10	111.90 (9)	O5—C15—C16	109.15 (10)
C9—O4—H4O	107.3 (15)	C14—C15—C16	103.16 (10)
C15—O5—H5O	106.4 (15)	O5—C15—H15	109.9
C22—O6—H6O	107.0 (15)	C14—C15—H15	109.9
C26—O7—C23	108.81 (9)	C16—C15—H15	109.9
O1—C1—C2	109.52 (10)	C15—C16—C17	106.43 (9)
O1—C1—C10	105.16 (9)	C15—C16—H16A	110.4
C2—C1—C10	105.12 (9)	C17—C16—H16A	110.4
O1—C1—H1	112.2	C15—C16—H16B	110.4
C2—C1—H1	112.2	C17—C16—H16B	110.4
C10—C1—H1	112.2	H16A—C16—H16B	108.6
C3—C2—C1	104.35 (9)	C13—C17—C16	103.05 (9)
C3—C2—H2A	110.9	C13—C17—C20	116.97 (10)
C1—C2—H2A	110.9	C16—C17—C20	114.87 (9)
C3—C2—H2B	110.9	C13—C17—H17	107.1
C1—C2—H2B	110.9	C16—C17—H17	107.1
H2A—C2—H2B	108.9	C20—C17—H17	107.1
O3—C3—O2	120.86 (11)	C14—C18—H18A	109.5
O3—C3—C2	128.16 (11)	C14—C18—H18B	109.5
O2—C3—C2	110.98 (10)	H18A—C18—H18B	109.5
O1—C4—C30	107.32 (10)	C14—C18—H18C	109.5
O1—C4—C29	109.20 (11)	H18A—C18—H18C	109.5
C30—C4—C29	110.52 (10)	H18B—C18—H18C	109.5
O1—C4—C5	102.53 (9)	C10—C19—C9	122.19 (10)
C30—C4—C5	112.86 (11)	C10—C19—H19A	106.8
C29—C4—C5	113.86 (10)	C9—C19—H19A	106.8
C6—C5—C10	117.30 (9)	C10—C19—H19B	106.8
C6—C5—C4	114.91 (9)	C9—C19—H19B	106.8
C10—C5—C4	103.33 (9)	H19A—C19—H19B	106.6
C6—C5—H5A	106.9	C21—C20—C22	111.17 (10)
C10—C5—H5A	106.9	C21—C20—C17	112.76 (9)
C4—C5—H5A	106.9	C22—C20—C17	108.63 (9)
C5—C6—C7	114.73 (10)	C21—C20—H20	108.0
C5—C6—H6A	108.6	C22—C20—H20	108.0
C7—C6—H6A	108.6	C17—C20—H20	108.0
C5—C6—H6B	108.6	C20—C21—H21A	109.5
C7—C6—H6B	108.6	C20—C21—H21B	109.5

Table S3. *Cont.*

H6A—C6—H6B	107.6	H21A—C21—H21B	109.5
C8—C7—C6	113.61 (10)	C20—C21—H21C	109.5
C8—C7—H7A	108.8	H21A—C21—H21C	109.5
C6—C7—H7A	108.8	H21B—C21—H21C	109.5
C8—C7—H7B	108.8	O6—C22—C23	110.54 (9)
C6—C7—H7B	108.8	O6—C22—C20	112.73 (9)
H7A—C7—H7B	107.7	C23—C22—C20	114.44 (10)
C9—C8—C7	113.37 (9)	O6—C22—H22	106.2
C9—C8—C14	114.47 (9)	C23—C22—H22	106.2
C7—C8—C14	112.83 (9)	C20—C22—H22	106.2
C9—C8—H8	105.0	O7—C23—C24	103.92 (9)
C7—C8—H8	105.0	O7—C23—C22	109.39 (9)
C14—C8—H8	105.0	C24—C23—C22	114.25 (11)
O4—C9—C8	107.50 (9)	O7—C23—H23	109.7
O4—C9—C11	109.48 (10)	C24—C23—H23	109.7
C8—C9—C11	108.71 (9)	C22—C23—H23	109.7
O4—C9—C19	111.42 (9)	C25—C24—C23	111.10 (11)
C8—C9—C19	113.07 (9)	C25—C24—H24	124.5
C11—C9—C19	106.60 (9)	C23—C24—H24	124.5
O2—C10—C19	109.50 (9)	C24—C25—C26	106.24 (11)
O2—C10—C5	108.66 (9)	C24—C25—C27	131.48 (13)
C19—C10—C5	118.97 (9)	C26—C25—C27	122.27 (12)
O2—C10—C1	103.80 (8)	O8—C26—O7	121.02 (12)
C19—C10—C1	110.80 (10)	O8—C26—C25	129.10 (13)
C5—C10—C1	103.97 (9)	O7—C26—C25	109.87 (10)
C12—C11—C9	111.61 (10)	C25—C27—H27A	109.5
C12—C11—H11A	109.3	C25—C27—H27B	109.5
C9—C11—H11A	109.3	H27A—C27—H27B	109.5
C12—C11—H11B	109.3	C25—C27—H27C	109.5
C9—C11—H11B	109.3	H27A—C27—H27C	109.5
H11A—C11—H11B	108.0	H27B—C27—H27C	109.5
C13—C12—C11	122.33 (10)	C4—C29—H29A	109.5
C13—C12—H12	118.8	C4—C29—H29B	109.5
C11—C12—H12	118.8	H29A—C29—H29B	109.5
C12—C13—C14	123.17 (10)	C4—C29—H29C	109.5
C12—C13—C17	126.10 (10)	H29A—C29—H29C	109.5
C14—C13—C17	109.75 (9)	H29B—C29—H29C	109.5
C13—C14—C18	109.38 (9)	C4—C30—H30A	109.5
C13—C14—C15	100.05 (9)	C4—C30—H30B	109.5
C18—C14—C15	107.04 (10)	H30A—C30—H30B	109.5
C13—C14—C8	114.24 (9)	C4—C30—H30C	109.5
C18—C14—C8	106.86 (9)	H30A—C30—H30C	109.5
C15—C14—C8	118.84 (10)	H30B—C30—H30C	109.5
C4—O1—C1—C2	151.82 (10)	C12—C13—C14—C8	4.53 (17)
C4—O1—C1—C10	39.32 (11)	C17—C13—C14—C8	−164.78 (9)
O1—C1—C2—C3	−94.37 (11)	C9—C8—C14—C13	23.20 (14)
C10—C1—C2—C3	18.16 (12)	C7—C8—C14—C13	154.85 (10)
C10—O2—C3—O3	178.19 (11)	C9—C8—C14—C18	144.31 (10)
C10—O2—C3—C2	−1.70 (13)	C7—C8—C14—C18	−84.04 (12)
C1—C2—C3—O3	169.21 (12)	C9—C8—C14—C15	−94.59 (12)
C1—C2—C3—O2	−10.91 (13)	C7—C8—C14—C15	37.06 (13)
C1—O1—C4—C30	−164.02 (10)	C13—C14—C15—O5	−77.42 (11)
C1—O1—C4—C29	76.16 (11)	C18—C14—C15—O5	168.58 (10)
C1—O1—C4—C5	−44.93 (12)	C8—C14—C15—O5	47.58 (14)
O1—C4—C5—C6	161.03 (10)	C13—C14—C15—C16	41.04 (10)
C30—C4—C5—C6	−83.84 (13)	C18—C14—C15—C16	−72.96 (11)
C29—C4—C5—C6	43.21 (15)	C8—C14—C15—C16	166.04 (9)

Table S3. *Cont.*

O1—C4—C5—C10	32.01 (11)	O5—C15—C16—C17	89.64 (12)
C30—C4—C5—C10	147.14 (10)	C14—C15—C16—C17	−32.45 (12)
C29—C4—C5—C10	−85.81 (12)	C12—C13—C17—C16	−151.90 (13)
C10—C5—C6—C7	−53.31 (15)	C14—C13—C17—C16	17.02 (12)
C4—C5—C6—C7	−175.02 (10)	C12—C13—C17—C20	81.06 (15)
C5—C6—C7—C8	90.62 (13)	C14—C13—C17—C20	−110.02 (11)
C6—C7—C8—C9	−74.96 (12)	C15—C16—C17—C13	10.00 (12)
C6—C7—C8—C14	152.85 (9)	C15—C16—C17—C20	138.36 (10)
C7—C8—C9—O4	−65.08 (12)	O2—C10—C19—C9	−56.78 (13)
C14—C8—C9—O4	66.31 (12)	C5—C10—C19—C9	68.95 (14)
C7—C8—C9—C11	176.50 (10)	C1—C10—C19—C9	−170.68 (9)
C14—C8—C9—C11	−52.12 (13)	O4—C9—C19—C10	48.78 (14)
C7—C8—C9—C19	58.32 (13)	C8—C9—C19—C10	−72.44 (13)
C14—C8—C9—C19	−170.29 (9)	C11—C9—C19—C10	168.16 (10)
C3—O2—C10—C19	−105.16 (10)	C13—C17—C20—C21	−35.50 (13)
C3—O2—C10—C5	123.40 (10)	C16—C17—C20—C21	−156.51 (10)
C3—O2—C10—C1	13.20 (11)	C13—C17—C20—C22	−159.19 (9)
C6—C5—C10—O2	113.27 (11)	C16—C17—C20—C22	79.80 (12)
C4—C5—C10—O2	−119.19 (9)	C21—C20—C22—O6	170.75 (9)
C6—C5—C10—C19	−12.86 (15)	C17—C20—C22—O6	−64.62 (12)
C4—C5—C10—C19	114.68 (11)	C21—C20—C22—C23	43.29 (12)
C6—C5—C10—C1	−136.65 (11)	C17—C20—C22—C23	167.92 (9)
C4—C5—C10—C1	−9.11 (11)	C26—O7—C23—C24	−1.48 (13)
O1—C1—C10—O2	96.47 (10)	C26—O7—C23—C22	120.92 (11)
C2—C1—C10—O2	−19.11 (11)	O6—C22—C23—O7	−65.49 (12)
O1—C1—C10—C19	−146.09 (9)	C20—C22—C23—O7	63.08 (13)
C2—C1—C10—C19	98.33 (11)	O6—C22—C23—C24	50.51 (12)
O1—C1—C10—C5	−17.15 (11)	C20—C22—C23—C24	179.07 (10)
C2—C1—C10—C5	−132.73 (10)	O7—C23—C24—C25	−0.03 (15)
O4—C9—C11—C12	−62.02 (13)	C22—C23—C24—C25	−119.16 (12)
C8—C9—C11—C12	55.15 (13)	C23—C24—C25—C26	1.39 (16)
C19—C9—C11—C12	177.35 (10)	C23—C24—C25—C27	−177.72 (15)
C9—C11—C12—C13	−30.64 (18)	C23—O7—C26—O8	−177.88 (13)
C11—C12—C13—C14	−0.4 (2)	C23—O7—C26—C25	2.39 (14)
C11—C12—C13—C17	167.14 (12)	C24—C25—C26—O8	177.91 (15)
C12—C13—C14—C18	−115.18 (13)	C27—C25—C26—O8	−2.9 (2)
C17—C13—C14—C18	75.52 (12)	C24—C25—C26—O7	−2.38 (15)
C12—C13—C14—C15	132.63 (12)	C27—C25—C26—O7	176.82 (13)
C17—C13—C14—C15	−36.68 (11)		

Table S4. Anisotropic displacement parameters (Å2) for micrandilactone H.

	U^{11}	U^{22}	U^{33}	U^{12}	U^{13}	U^{23}
O1	0.0117 (3)	0.0197 (5)	0.0113 (3)	0.0010 (3)	0.0061 (3)	0.0015 (3)
O2	0.0112 (3)	0.0092 (4)	0.0087 (3)	0.0020 (3)	0.0032 (3)	0.0007 (3)
O3	0.0168 (4)	0.0127 (4)	0.0117 (4)	0.0004 (3)	0.0011 (3)	0.0004 (3)
O4	0.0121 (3)	0.0169 (4)	0.0092 (3)	0.0042 (3)	0.0031 (3)	0.0013 (3)
O5	0.0283 (5)	0.0116 (4)	0.0194 (4)	0.0058 (4)	0.0138 (4)	0.0036 (3)
O6	0.0136 (4)	0.0137 (4)	0.0135 (4)	0.0018 (3)	0.0059 (3)	0.0007 (3)
O7	0.0115 (3)	0.0179 (5)	0.0110 (3)	0.0004 (3)	0.0038 (3)	0.0032 (3)
O8	0.0258 (5)	0.0403 (7)	0.0103 (4)	−0.0099 (5)	0.0037 (4)	0.0006 (4)
C1	0.0129 (4)	0.0124 (5)	0.0117 (4)	0.0027 (4)	0.0065 (4)	0.0002 (4)
C2	0.0155 (5)	0.0133 (5)	0.0106 (4)	0.0012 (4)	0.0057 (4)	−0.0017 (4)
C3	0.0115 (4)	0.0104 (5)	0.0100 (4)	−0.0019 (4)	0.0039 (4)	−0.0012 (4)
C4	0.0105 (4)	0.0166 (6)	0.0117 (4)	0.0027 (4)	0.0049 (4)	0.0030 (4)
C5	0.0084 (4)	0.0121 (5)	0.0107 (4)	0.0002 (4)	0.0039 (3)	0.0006 (4)
C6	0.0092 (4)	0.0193 (6)	0.0096 (4)	−0.0004 (4)	0.0032 (3)	0.0003 (4)
C7	0.0122 (4)	0.0133 (5)	0.0121 (4)	−0.0037 (4)	0.0058 (4)	−0.0037 (4)
C8	0.0095 (4)	0.0094 (5)	0.0092 (4)	−0.0002 (4)	0.0042 (3)	−0.0005 (4)
C9	0.0113 (4)	0.0088 (5)	0.0094 (4)	−0.0008 (4)	0.0047 (3)	−0.0011 (4)
C10	0.0096 (4)	0.0087 (4)	0.0093 (4)	0.0021 (3)	0.0040 (3)	0.0014 (3)
C11	0.0206 (5)	0.0140 (6)	0.0159 (5)	−0.0074 (4)	0.0119 (4)	−0.0060 (4)
C12	0.0146 (5)	0.0151 (5)	0.0137 (5)	−0.0063 (4)	0.0083 (4)	−0.0040 (4)
C13	0.0108 (4)	0.0104 (5)	0.0101 (4)	−0.0007 (4)	0.0051 (3)	−0.0001 (4)
C14	0.0101 (4)	0.0096 (5)	0.0082 (4)	−0.0002 (3)	0.0037 (3)	0.0000 (3)
C15	0.0162 (5)	0.0103 (5)	0.0137 (5)	−0.0007 (4)	0.0086 (4)	−0.0018 (4)
C16	0.0184 (5)	0.0115 (5)	0.0167 (5)	−0.0004 (4)	0.0122 (4)	−0.0021 (4)
C17	0.0103 (4)	0.0120 (5)	0.0096 (4)	0.0009 (4)	0.0048 (3)	0.0005 (4)
C18	0.0124 (4)	0.0210 (6)	0.0103 (4)	0.0016 (4)	0.0042 (4)	0.0018 (4)
C19	0.0132 (4)	0.0082 (5)	0.0114 (4)	0.0001 (4)	0.0067 (4)	−0.0007 (4)
C20	0.0091 (4)	0.0124 (5)	0.0090 (4)	0.0015 (4)	0.0038 (3)	0.0013 (4)
C21	0.0176 (5)	0.0127 (5)	0.0149 (5)	0.0022 (4)	0.0087 (4)	0.0027 (4)
C22	0.0092 (4)	0.0142 (5)	0.0095 (4)	0.0002 (4)	0.0039 (3)	0.0002 (4)
C23	0.0098 (4)	0.0157 (5)	0.0091 (4)	−0.0013 (4)	0.0033 (3)	−0.0010 (4)
C24	0.0109 (4)	0.0202 (6)	0.0150 (5)	0.0000 (4)	0.0071 (4)	0.0016 (4)
C25	0.0178 (5)	0.0175 (6)	0.0161 (5)	−0.0017 (4)	0.0113 (4)	−0.0001 (4)
C26	0.0185 (5)	0.0181 (6)	0.0115 (4)	−0.0064 (4)	0.0068 (4)	−0.0006 (4)
C27	0.0359 (8)	0.0304 (8)	0.0267 (7)	0.0028 (6)	0.0247 (6)	0.0001 (6)
C29	0.0144 (5)	0.0182 (6)	0.0193 (5)	0.0058 (4)	0.0082 (4)	0.0045 (5)
C30	0.0115 (5)	0.0223 (6)	0.0172 (5)	0.0009 (4)	0.0063 (4)	0.0054 (5)

Table S5. Hepatoprotective effect of micrandilactone H (10 μM) against APAP-induced toxicity in HepG2 cell.

**Compounds**	**OD (mean ± SD)**	**Cell Survival Rate (% of Normal)**
Control	1.286 ± 0.147	100.00
APAP 8 mM	0.494 ± 0.111 ***	38.40
Micrandilactone H	0.725 ± 0.042 ##	56.84
Bicylol	0.678 ± 0.020 ##	53.16

*** $p < 0.001$, compared with control; ## $p < 0.01$, compared with model (APAP)
